# Perforation of the sigmoid colon with formation of a sigmoido-ureteral fistula caused by an ingested denture: a case report

**DOI:** 10.1093/jscr/rjaf041

**Published:** 2025-02-10

**Authors:** Nina Schraps, Joelle C Korte, Antonie Willner, Thilo Hackert, Nathaniel Melling, Baris Mercanoglu

**Affiliations:** Department of General, Visceral and Thoracic Surgery, University Medical Center Hamburg-Eppendorf, Martinistraße 52, 20246 Hamburg, Germany; Department of General, Visceral and Thoracic Surgery, University Medical Center Hamburg-Eppendorf, Martinistraße 52, 20246 Hamburg, Germany; Department of General, Visceral and Thoracic Surgery, University Medical Center Hamburg-Eppendorf, Martinistraße 52, 20246 Hamburg, Germany; Department of General, Visceral and Thoracic Surgery, University Medical Center Hamburg-Eppendorf, Martinistraße 52, 20246 Hamburg, Germany; Department of General, Visceral and Thoracic Surgery, University Medical Center Hamburg-Eppendorf, Martinistraße 52, 20246 Hamburg, Germany; Department of General, Visceral and Thoracic Surgery, University Medical Center Hamburg-Eppendorf, Martinistraße 52, 20246 Hamburg, Germany

**Keywords:** denture, sigmoido-ureteral fistula, perforation, low anterior resection

## Abstract

Accidentally ingested objects such as dentures can have serious health consequences with damage to the gastrointestinal tract. We report the case of a patient who ingested her dental plate unnoticed, leading to a sigmoid perforation and formation of a sigmoido-ureteral fistula. The patient presented to our emergency department with symptoms 3 months after ingestion. After unsuccessful endoscopic retrieval, a low anterior rectal resection with retrieval of the swallowed denture and resection of the left ureter with subsequent reconstruction surgery was performed.

## Introduction

Besides different foods, dentures are among the most commonly ingested foreign bodies in adults accounting for 4%–18% of cases [1, 2]. Over 80% of swallowed foreign bodies pass through the gastrointestinal tract without intervention and surgical treatment is required in ˂1% of cases [1, 3]. Most objects typically retain in the upper gastrointestinal tract [2]. Complications due to ingested foreign bodies include obstruction and impactions [4], gastrointestinal bleeding [2] and fistula formation [5]. Perforation caused by swallowed foreign bodies occurs in ˂1% of cases and are most common in the terminal ileum followed by the rectosigmoid junction [5, 6].

## Case report

We report the case of a 59-year-old female patient who was referred to our emergency department in February 2023 due to sonographic findings of a mass in the left lower abdomen. The patient reported on discoloration of the urine, altered bowel habits and lower abdominal pain. The patient further noted a loss of appetite accompanied by a weight loss of 14 kg within the last two months. She did not report any fever, night sweat, rectal bleeding, nausea, or vomiting. Clinical examination revealed supravesical pressure pain with otherwise normal findings.

**Figure 1:**
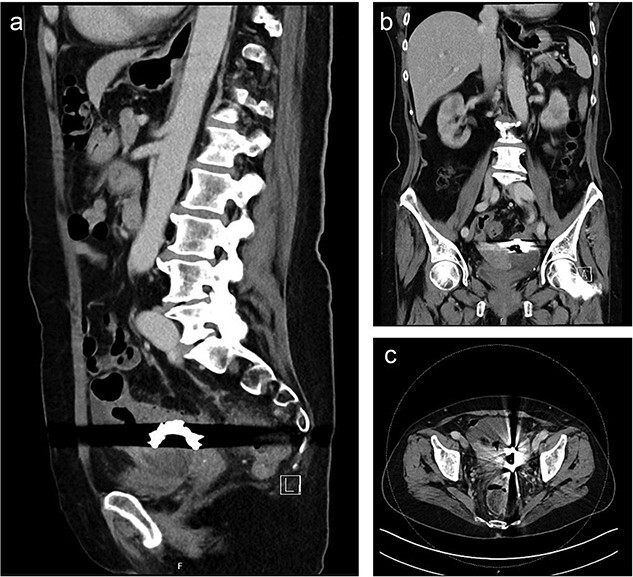
Sagittal (a), coronal (b) and axial slice (c) of a preoperative CT scan showing the ingested denture in the sigmoid colon with adjacent fluid collection and vesical air inclusions.

**Figure 2:**
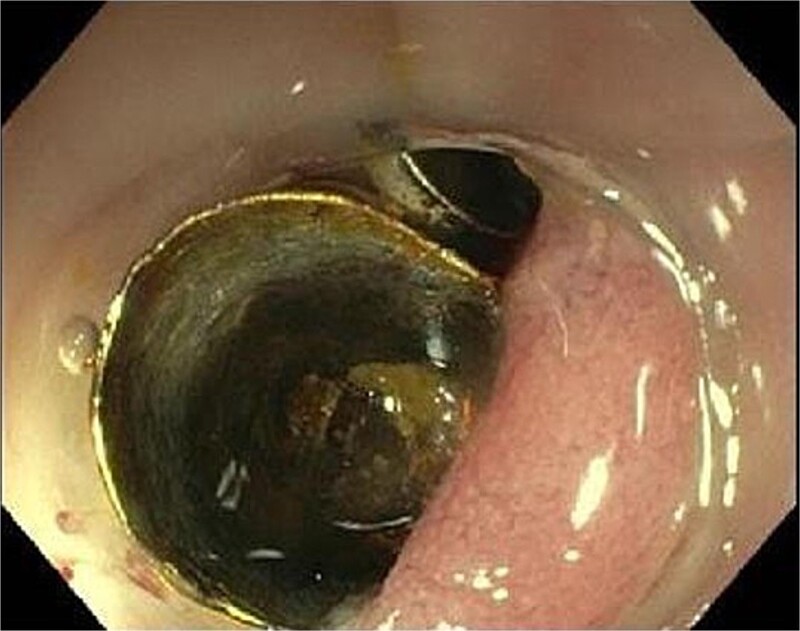
Endoscopic view showing the metal denture at 35 cm from anal verge with swollen and scarred stenosis of the sigmoid colon.

Computed tomography (CT) showed a foreign body in the sigmoid colon with adjacent fluid collection (Fig. 1). Due to vesical air inclusions, a sigmoido-vesical fistula was suspected. When further asked, the patient recalled that she had lost her denture 3 months ago at the Christmas market in December 2022. Colonoscopy revealed the impacted foreign body at 35 cm from anal verge with swollen and scarred stenosis of the sigmoid colon (Fig. 2). Endoscopic retrieval of the metal denture was not possible.

Emergency exploratory laparotomy revealed a severe inflammatory conglomerate tumor involving the sigmoid colon, rectum, urinary bladder, uterus, left adnexa, and perforation into the left ureter. A low anterior rectal resection en bloc with resection of the left adnexa, tube and ureter was performed followed by the creation of a colostomy of the descending colon and an ureterocutaneostomy. This was followed one week later by an early reconnection operation with establishment of bowel continuity in the sense of a descendorectostomy. And finally in December 2023, the patient underwent reconstruction of the left ureter with interposition of ileum. At the last follow-up in September 2024 the patient presented with no complaints.

## Discussion

While most ingested foreign bodies pass through the gastrointestinal tract without any necessary interventions, they can be associated with serious complications or even mortality [1]. The majority of accidentally swallowed objects are found in children [7]. Risk factors for foreign body ingestion in adulthood include psychiatric disorders, edentulousness, alcohol consumption, or intended ingestion due to a suspected secondary gain [7, 8].

Factors that should be considered in diagnosis and therapy decisions include the type of object, the time since ingestion, the anatomical localization and the clinical presentation [9]. Initially the foreign body should be localized and identified based on the medical history, physical examination and imaging [1, 4].

Possible complications of foreign body ingestion include gastrointestinal obstruction and bleeding, fistula formation, perforation or extra luminal migration [7, 10]. Impactions usually occur in areas of physiological narrowing, especially in the esophagus, depending on the shape and size of the ingested object [4]. Patients with previous abdominal surgery or congenital malformation are at higher risk for impaction [11].

According to literature most patients present within the first 24 h [4]. Our patient was referred to our emergency department almost 3 months after the incident, which was previously reported to be associated with a greater risk of complications [11]. The chance of swallowed objects spontaneously passing through the gastrointestinal tract becomes increasingly smaller after 24 h and is linked to an increased risk of complications [11, 12]. As in the case of our patient there are furthermore complications secondary to perforation [5], including previous reports on enteroenteric [13] and enterovascular fistula [14], abscesses of the liver [15] or brain [12], as well as septic cases [16].

If the objects cannot be retrieved endoscopically, the decision on surgical treatment depends on several factors and is required in less than 1% of cases [1]. Perforation has previously been proposed as the only absolute indication [1]. Other factors to consider include the time since the foreign body was swallowed, signs of peritonitis or bowel obstruction, detection of a fistula and other complications that cannot be resolved endoscopically [1, 11].

In summary, the early identification of the foreign body, risk stratification and, if necessary, early intervention can help avoid complications such as perforation or fistula formation.
